# Downstream Targets of Cyclic Nucleotides in Plants

**DOI:** 10.3389/fpls.2018.01428

**Published:** 2018-10-01

**Authors:** Brygida Świeżawska, Maria Duszyn, Krzysztof Jaworski, Adriana Szmidt-Jaworska

**Affiliations:** Chair of Plant Physiology and Biotechnology, Faculty of Biology and Environmental Protection, Nicolaus Copernicus University, Torun, Poland

**Keywords:** cyclic AMP, cyclic GMP, cyclic nucleotide effectors, phosphodiesterases, cNMP-dependent protein kinases, plants, signaling

## Abstract

Efficient integration of various external and internal signals is required to maintain adaptive cellular function. Numerous distinct signal transduction systems have evolved to allow cells to receive these inputs, to translate their codes and, subsequently, to expand and integrate their meanings. Two of these, cyclic AMP and cyclic GMP, together referred to as the cyclic nucleotide signaling system, are between them. The cyclic nucleotides regulate a vast number of processes in almost all living organisms. Once synthesized by adenylyl or guanylyl cyclases, cyclic nucleotides transduce signals by acting through a number of cellular effectors. Because the activities of several of these effectors are altered simultaneously in response to temporal changes in cyclic nucleotide levels, agents that increase cAMP/cGMP levels can trigger multiple signaling events that markedly affect numerous cellular functions. In this mini review, we summarize recent evidence supporting the existence of cNMP effectors in plant cells. Specifically, we highlight cAMP-dependent protein kinase A (PKA), cGMP-dependent kinase G (PKG), and cyclic nucleotide phosphodiesterases (PDEs). Essentially this manuscript documents the progress that has been achieved in recent decades in improving our understanding of the regulation and function of cNMPs in plants and emphasizes the current gaps and unanswered questions in this field of plant signaling research.

## Introduction

“Signaling” is defined as a set of events occurring between the perception of a signal and the appearance of a measurable change in the organism, on a broad time scale (Quail, 2006).

cNMPs, such as adenosine-3′,5′-cyclic monophosphate (cAMP) and guanosine-3′,5′-cyclic monophosphate (cGMP), are involved in signal transduction in all living cells. cNMPs are formed by purine nucleotide cyclases (NCs) from nucleotide triphosphate precursors (NTPs). Both the frequency (amplitude and duration) and location of changes in the levels of cNMPs depend on the activity of cNMP PDEs, which catalyze cNMP hydrolysis. Consequently, an increase in intracellular cNMP levels affects the activity of downstream effectors, particularly protein kinases, ion channels, and transcription factors, enabling the divergence of cNMP signals.

**FIGURE 1:**
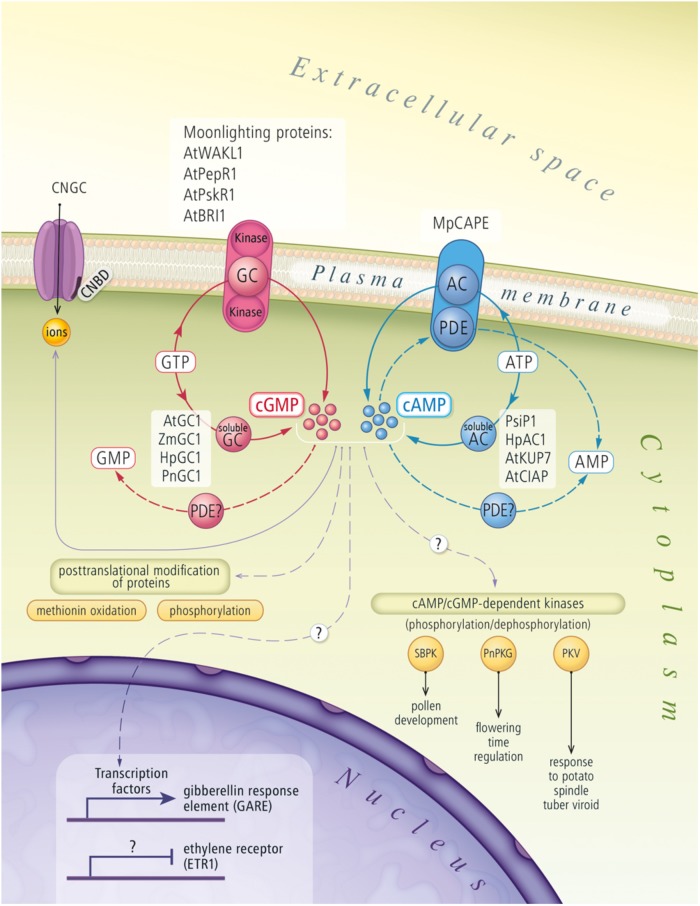
Cyclic nucleotides signaling pathway in plant cells. The figure draws on actual knowledge about biosynthesis and signal transduction of cNMPs in plants. AC, adenylyl cyclase; AMP, adenosine monophosphate; AtBRI1, *A. thaliana* brassinosteroid receptor; AtClAP, *A. thaliana* clathrin assembly protein; AtGC1, *A. thaliana* guanylyl cyclase; AtKUP7, *A. thaliana* K^+^-uptake permease 7; ATP, adenosine triphosphate; AtPepR1, *A. thaliana* peptide signaling molecule (Pep1) receptor; AtPSKR1, *A. thaliana* phytosulfokine receptor 1; AtWAKL1, *A. thaliana* stress-responsive wall-associated kinase-like molecule; cAMP, cyclic adenosine monophosphate; cGMP, cyclic guanosine monophosphate; CNBD, cyclic nucleotide binding domain; CNGC, cyclic nucleotide gated channel; GC, guanylyl cyclase; GMP, guanosine monophosphate; GTP, guanosine triphosphate; HpAC1, *Hippeastrum hybridum* adenylyl cyclase 1; HpGC1, *Hippeastrum hybridum* guanylyl cyclase 1; MpCAPE, *Marchantia polymorpha* combined AC with PDE; PDE, phosphodiesterase; PKV, protein kinase viroid induced protein; PnGC1, *Pharbitis nil* guanylyl cyclase 1; PnPKG, *Pharbitis nil* cyclic GMP-dependent protein kinase; PsiP1, pollen signaling protein with adenylyl cyclase activity; SBPK, *Solanum berthaultii* protein kinase; ZmGC1, *Zea mays* guanylyl cyclase 1.

Recently, studies characterizing individual elements and signal transduction pathways have been used to describe this intracellular network in plants. A schematic depicting the current understanding of the cNMP signaling system in plant cells is summarized in **Figure 1**. Nonetheless, we are still far from obtaining a complete understanding of the functions of these systems. The roles and biosynthesis of cNMPs in plants have been discussed for many years (Brown et al., 1977, 1979; Kato et al., 1983; Schuurink et al., 1998; Gehring, 2010; Gehring and Turek, 2017). Therefore, the improvements in and availability of techniques that are sufficiently sensitive to quantify cNMPs have enabled researchers to unambiguously confirm the existence of cNMPs in plant cells.

This mini review aims to provide an update on the progress in our understanding of cNMP-dependent downstream signaling in plants. This progress is steady, but quite slow probably due to the lack of structural similarity between animal, bacterial and plant cNMP-dependent proteins. A classic database search of plant counterparts of animal and bacterial cNMP-binding elements was unsuccessful. This situation has forced the creation of new methods, particularly bioinformatics tools, facilitating the identification of a list of candidate cNMP effectors. The best known and most extensively studied group of cNMP targets is a family of CNGCs. Several excellent reviews on plant CNGCs exist (Kaplan et al., 2007; Moeder et al., 2011; Rehman, 2014; Jha et al., 2016), and hence, their structure and function will not be further elaborated here. We summarize how recent evidence supports the existence of other cNMP effectors in plant cells. Specifically, we highlight PKA, PKG, and cNMP PDEs.

## cNmp Scavengers (3′,5′-cNmp Pdes) in Plants

PDEs are the only enzymes that catalyze the hydrolysis of cAMP and cGMP to inactive AMP and GMP, respectively. This deactivation plays an important role in intracellular signaling. Based on sequence and structural similarities, the PDE superfamily was classified into three groups (classes I–III) (Conti and Beavo, 2007). Although each group has a different sequence and structure, they still utilize the same mechanism for cNMP hydrolysis. Mammalian PDEs and certain high and low eukaryotic PDEs are class I PDEs. This class is divided into 11 families (Bender and Beavo, 2006). PDEs 1, 2, 3, 10, and 11 are dual-substrate enzymes that hydrolyze cAMP and cGMP; PDEs 4, 7, and 8 are cAMP-specific enzymes; and PDEs 5, 6, and 9 are cGMP-specific enzymes (Lugnier, 2006). Animal PDEs are good drugs targets and have great pharmacological potential and commercial value, making them an ideal research targets (Corbin, 2004). The knowledge of these proteins has achieved substantial progress over the last 55 years and is completely incomparable with others living organisms such as plants or bacteria (Bender and Beavo, 2006; Matange, 2015; Gross and Durner, 2016). Class II PDEs are expressed in some lower eukaryotes and bacteria, whereas class III PDEs are only detected in prokaryotes (Conti and Beavo, 2007; Matange, 2015; Gross and Durner, 2016).

Although the role of cNMPs has been documented in many physiological processes in plants, the knowledge about its hydrolysis is actually limited only to classical biochemical experiments not providing definitive conclusions. In higher plants, PDEs are divided into one of two groups: (1) PDEs responsible for the inactivation of 2′,3′-cyclic nucleotides (2′,3′-cNMPs) or (2) the inactivation of 3′,5′-cyclic nucleotides (3′,5′-cNMPs). The first putative PDEs shown to catalyze the hydrolysis of 2′,3′-cNMP to 2′-NMP were originally purified from *Triticum aestivum* germ (Tyc et al., 1987), and these PDEs were subsequently proposed to be involved in tRNA splicing (Culver et al., 1994). Another PDE was identified in *Arabidopsis thaliana*, and the properties of the recombinant protein were similar to that of the PDE from *T. aestivum*. Moreover, both PDEs hydrolyzed 2′,3′-cNMP to 2′-NMP (Genschik et al., 1997).

Another group of PDEs are 3′,5′-cNMP PDEs. This enzyme hydrolyses 3′,5′-cNMPs to produce a mixture of 3′-NMP and 5′-NMP. The first PDE that was able to hydrolyze both 2′,3′-cNMP and 3′,5′-cNMP was isolated and partially purified from *Pisum sativum* seedlings. This enzyme catalyzes the hydrolysis of 2′,3′-cAMP to 3′-AMP, whereas 3′,5′-cAMP is hydrolyzed to a mixture of 3′-AMP and 5′-AMP (Lin and Varner, 1972). Thus, in contrast to their animal counterparts, which possess a single activity, potential plant PDEs appear to have a dual enzymatic function. Enzymes with these activities have been identified in *Solanaceae* (Ashton and Polya, 1975; Matsuzaki and Hashimoto, 1981; Zan-Kowalczewska et al., 1984; Abel et al., 2000) and *Fabaceae* (Lin and Varner, 1972). Surprisingly, until recently, potential plant PDE activity has only been detected in protein extracts (Ashton and Polya, 1975; Zan-Kowalczewska et al., 1984). Ammonium sulfate precipitation, CM-cellulose chromatography, gel filtration chromatography, and gel permeation chromatography have not been sufficient for the purification of PDEs and the determination of their biochemical properties. Plant PDEs were postulated to form a complex with other enzymes, which may impede their purification, extraction and characterization (Gross and Durner, 2016), especially since monomeric and tetrameric forms have been observed for the PDE from *S. tuberosum*. Analyses using gel filtration and sucrose density gradient centrifugation led to the determination of a molecular weight of 79–81 kDa for the PDE monomer and 343–346 kDa for the PDE tetramer (Zan-Kowalczewska et al., 1984). The authors suggested that these two forms undoubtedly correspond to the *S. tuberosum* cNMP PDE classes I and II, which have a molecular weights of 240 and 80 kDa, respectively (Ashton and Polya, 1975). However, PDEs could form complexes with acid phosphatases, ribonucleases, nucleotidases, and ATPases (Brown et al., 1980). A more in-depth understanding of PDE complexity in plant cells will be obtained after the identification of different *PDE* genes. However, the field is evolving very slowly, if at all. The first and only molecularly confirmed PDE in liverwort *Marchantia polymorpha* is named MpCAPE (Kasahara et al., 2016). Biochemical analyses of the recombinant protein showed that MpCAPE exhibits both AC and PDE activities. This protein has an AC domain located at its C-terminus and a cNMP PDE domain at its N-terminus. The PDE domain of MpCAPE is similar to the catalytic domain of human PDE enzymes and possesses all characteristic amino acid residues required to bind Zn^2+^ and Mg^2+^. According to bioinformatics analyses, the sequence of this PDE domain was not detected within other higher plant proteins. The identification and full molecular and biochemical characterization of enzymes involved in cNMPs inactivation is a challenge for the future plant signaling research.

## cNmp-Dependent Protein Kinases in Plants

Based on our knowledge of cNMP-dependent protein kinases, PKA and PKG in animals, should be the main enzymes that decode the cNMP signal and then activate target proteins by phosphorylation (Scott, 1991). However, although some indirect evidence exists for enzyme activation by cNMPs in plants, the existence of plant cNMP-dependent protein kinases is still controversial. Similar to plant PDEs, these proteins have very low sequence, structural and biochemical homology with their fungal and animal counterparts. In addition, the substrate specificity of plant PKA/PKG isoforms may be different from kinases derived from other kingdoms, which impedes the use of routine biochemical experiments designed to examine substrates for mammalian kinases, such as kemptide or glasstide (Bridges et al., 2005). Therefore, the cNMP signaling pathway in plants has likely evolved differently from that in mammals (Bridges et al., 2005). In the early 1980s, three putative cAMP-responsive protein kinases were identified in *Lemna paucicostata* and were shown to catalyze the phosphorylation of histones *in vitro* (Kato et al., 1983). Later, PKA activity was detected in *Zea mays*, *Cocos nucifera* (Janistyn, 1988, 1989), *Petunia hybrid* var. Old Glory Blue (Polya et al., 1991), and *Oryza sativa* (Komatsu and Hirano, 1993). Moreover, cAMP-dependent phosphorylation of proteins localized to etioplasts of *Triticum vulgare* and stomatal cells of *Vicia faba* was reported (Newton and Smith, 2004). According to experiments performed in *Phaseolus vulgaris*, the level of cAMP-dependent phosphorylation increased after the application of forskolin (AC activator) or micromolar concentrations of cAMP and decreased after the application of a PKA inhibitor (Friedrich et al., 1999).

Some reports describing kinases regulated by cGMP in plants are also available. Rp-8-Br-cGMPS, an inhibitor of animal PKGs, prevented auxin-induced opening of stomatal cells in *Commelina communis* tissues, suggesting that potential PKG is expressed in this plant species (Cousson and Vavasseur, 1998). The activity of cGMP-binding proteins was also detected in crude homogenates of *Avena sativa* seedlings (Dubovskaya et al., 2002). Putative cGMP dependent kinase partially purified from tissues of *Pharbitis nil* was called PnPKG1 and cross-reacted with polyclonal antibodies raised against animal PKG. In addition, the enzyme activity was accelerated by low micromolar concentrations of cGMP, and histone H2B was preferentially phosphorylated as an exogenous substrate (Szmidt-Jaworska et al., 2003).

The reports providing indirect evidence for the presence of PKA/PKG in plant cells are mainly based on physiological experiments with using non-specific, inhibitors of these proteins in animals, so these results should be treated with caution and require molecular confirmation.

Surprisingly, although biochemical studies conducted on plant homogenates and partially purified protein fractions suggest the existence of cNMP-dependent phosphorylation in plants, molecular analyses are still lacking. To date, certain sequences in plant genomes have been identified as sharing homology with animal cNMP-dependent protein kinases. The *SBPK* gene, which was identified in *Solanum berthaultii*, encodes a protein with a motif that is characteristic of the catalytic subunit of cNMP-dependent protein kinases from yeasts and animals (Liu et al., 1999). *SBPK* does not contain any regulatory domains, indicating that *SBPK* probably exerts its function in combination with other regulatory subunits. A second gene called *PKV*, identified in *Lycopersicon esculentum*, was upregulated when the plant was infected with a severe strain of the potato spindle tuber viroid (PSTVd) (Hammond and Zhao, 2000). Bioinformatics analysis of the *PKV* coding region suggested that it is a serine/threonine protein kinase. This protein can undergo autophosphorylation *in vitro* on serine and tyrosine residues. Next, studies based on three-dimensional homology modeling revealed that a group of Pto-like kinases from *P. vulgaris* contains nearly all the characteristic structural features of cAMP-dependent protein kinase type α (cAPKα) (Vallad et al., 2001). An analysis of the sequence of a putative PKG (At2g20040) in *A. thaliana* showed 48% similarity to mammalian type II PKG, but the expression of several transcript variants in *Escherichia coli* cells resulted in insoluble and inactive proteins (Martinez-Atienza et al., 2007). Therefore, researchers have not yet determined whether this gene family encodes an active cNMP-dependent protein kinase.

More recently, genes encoding proteins with both GC and kinase domains called “moonlighting proteins” were identified in several plant genomes (Krupa et al., 2006; Irving et al., 2012). The characteristic feature of these proteins is a functional GC domain embedded within their intracellular kinase domain. This novel type of “moonlighting protein” is different from the animal counterparts, because their GC catalytic center is nested within the catalytic kinase domain and separated by linker sequences or domains. The first protein shown to be anchored in the cell membrane with both GC and kinase activities was postulated to be a brassinosteroid receptor in *A. thaliana* (AtBRI) (Kwezi et al., 2007). Further analyses identified other *A. thaliana* membrane proteins with dual GC-kinase activities: a stress-responsive wall-associated kinase-like molecule (AtWAKL10; At1g79680) (Meier et al., 2010), a peptide signaling molecule (Pep1) receptor (AtPepR1; At1g73080) (Qi et al., 2010) and phytosulfokine receptor 1 (AtPSKR1) (Kwezi et al., 2011). Both AtBRI1 and AtPSKR1 are dual tyrosine and serine/threonine kinases (Muleya et al., 2016; Oh et al., 2010). Currently, more than forty proteins with a similar domain architecture are believed to exist in the *A. thaliana* proteome (Wong and Gehring, 2013).

The production of cGMP may be due to the presence of endogenous factors that down-regulate kinase activity (Kwezi et al., 2011; Freihat et al., 2014). GC-kinases have been postulated to serve as a switch between downstream kinase-mediated and cGMP-mediated signaling cascades to elicit desired responses to particular stimuli (Freihat et al., 2014). According to the new mechanism of “moonlighting proteins” cGMP may be acting autonomously at the site where it is produced by regulating the kinase activity of the protein (Kwezi et al., 2018). Comparatively low levels of cGMP synthetized by the GC center in a highly localized region in response to a ligand binding are necessary to inhibit the kinase activity of this type of receptor.

The discovery of “moonlighting proteins” is very promising, however several less optimistic reports have been published. The GC activity of AtBRI1 was not confirmed in studies examining its crystal structure (Bojar et al., 2014). This activity of AtBRI1 was measured using HPLC, which is probably not sufficiently sensitive to detect the low levels of cGMP generated by plant GCs. Skepticism regarding the GC activity of another “moonlighting protein”, AtPepR1, was also expressed by Ashton (2011). The GC activity of AtPepR1 was described as “extraordinarily low” and the cGMP level was suggested to be an artifact or bacterial contamination, which might be biologically irrelevant. In summary, additionally studies are needed to improve our knowledge of the dual activity of “moonlighting proteins” and other cNMP-dependent kinases (Gross and Durner, 2016).

## Other Cyclic Nucleotide-Binding Proteins (CNBPs) and Cyclic Nucleotide-Dependent Proteins (CNDPs) in Plants

For several years, research has also been conducted to identify new and unusual downstream CNBPs. Twelve *A. thaliana* proteins were characterized as CNBPs, and their putative physiological roles in the photorespiration pathway and Calvin cycle were described. Moreover, a number of CNBP candidates were post-translationally modified by NO, transcriptionally co-expressed and functionally annotated to hydrogen peroxide signaling (H_2_O_2_) pathway and plant response to stress. The authors suggested that the newly identified CNBPs function together as a point of cross-talk between the cNMP, NO, and H_2_O_2_ signaling pathways that are activated during the plant defense response (Donaldson et al., 2016).

Other proteomic analyses revealed the cAMP-dependent changes in the *A. thaliana* proteome (Thomas et al., 2013; Alqurashi et al., 2016), particularly the activity of proteins with a role in light- and temperature-dependent responses (Thomas et al., 2013). Based on these results, the authors suggested that cAMP may function in light signaling, the regulation of photosynthesis and the response to temperature, similar to cyanobacteria, algae, and fungi. However, the mechanisms underlying these regulatory pathways remain unknown, which may be due to the binding of cAMP/cGMP to uncharacterized domains with low homology to well-known CNBD or GAF motifs (Thomas et al., 2013).

cNMPs are now known to act as regulators of specific elements in the promoters of genes, as shown for gibberellic acid- and ethylene-responsive genes (Bastian et al., 2010; Hussain et al., 2016). Moreover, cGMP appears to be involved in post-transcriptional modifications of proteins such as methionine oxidation (Marondedze et al., 2013) and phosphorylation (Isner et al., 2012).

Detailed structural studies of cNMP turnover or binding and of the various protein or nucleic acid effectors may help identify new classes of binding sites. These studies may also facilitate the rational design of molecules that inhibit or modulate cNMP action.

## Conclusion and Future Perspectives

Together with the universality of cNMP signaling, the diversity and differential expression of cNMP effectors make them important elements in cell signaling. Decades of work have refined some details of cAMP and cGMP signal transduction pathways in plants and have revealed an increasing number of areas in which these signaling events are important and even central contributors. Collectively, the data reported here reveal the complex mechanisms controlling cNMP, thus raising the question of the physiological relevance of these sophisticated mechanisms in plants. Current studies require the precise characterization of cyclases, cNMP scavengers, and different types of effectors. Clearly, cNMP is produced in multiple, discrete subcellular gradients that may carry information, thus indicating that the subcellular localizations of enzymes involved in cNMP metabolism are of critical importance. Over the next few years, our knowledge of cAMP and cGMP signaling in plant cells will continue to increase. Mathematical modeling of cNMP regulation may identify new mechanisms that are involved in the synthesis, action and degradation of cNMP. Firstly, the creation of new bioinformatics tools, such as the recently reported “GCPred” tool for predicting the GC functional center will significantly improve the search for new candidate cNMP-related proteins (Kwezi et al., 2018). Secondly, the development of a fast and non-complicated screening system for potential plant cNMP PDEs would facilitate a quick analysis of selected sequences with predicted PDE activity. In the case of ACs, screening test based on visual analysis of *E. coli* AC-deficient mutant colonies complemented by cloned plant ACs has routinely been performed (Świeżawska et al., 2014; Chatukuta et al., 2018). Similarly, complementation tests have also been used for bacterial and animal PDEs in mutant yeast strains deficient in *PDE* genes (Atienza and Colicelli, 1998; Alonso et al., 2007). To date, this screening tool has not been used for potential plant PDEs.

Therefore, new bioinformatics algorithms and molecular tools may provide opportunities to broaden our currently limited understanding of cNMP-dependent protein kinases and PDEs in plants. Ultimately, a better understanding of the function and interaction of these signaling pathways will enhance our appreciation of their contribution to the normal function of plants and their adaptations to unfavorable conditions.

However, the regulatory role of cNMP in plant cells may be based mainly on CNGC activation, and other cNMP sensors might play extremely marginal roles in this signaling pathway.

## Author Contributions

BŚ and MD wrote the manuscript. KJ and AS-J revised and critically evaluated the manuscript. All authors read and approved the manuscript.

## Conflict of Interest Statement

The authors declare that the research was conducted in the absence of any commercial or financial relationships that could be construed as a potential conflict of interest.

## Abbreviations

AC: adenylyl cyclase
AMP: adenosine monophosphate
ATP: adenosine triphosphate
cAMP: cyclic adenosine monophosphate
cGMP: cyclic guanosine monophosphate
CNBD: cyclic nucleotide biding domain
CNGC: cyclic nucleotide gated channel
cNMP: cyclic nucleotide
GAF: mammalian cGMP-binding PDEs, *Anabaena* adenylyl cyclases (ACs), and *E. coli* FhlA
GC: guanylyl cyclase
GMP: guanosine monophosphate
GTP: guanosine triphosphate
PDE: phosphodiesterase
PKA: cAMP-dependent protein kinase A
PKG: cGMP-dependent protein kinase G.
